# Annotated Pap cell images and smear slices for cell classification

**DOI:** 10.1038/s41597-024-03596-3

**Published:** 2024-07-07

**Authors:** David Kupas, Andras Hajdu, Ilona Kovacs, Zoltan Hargitai, Zita Szombathy, Balazs Harangi

**Affiliations:** 1 Department of Data Science and Visualization, Faculty of Informatics, University of Debrecen, Debrecen, Hungary; 2 Department of Pathology, Kenezy Gyula University Hospital and Clinic, University of Debrecen, Debrecen, Hungary

**Keywords:** Cancer screening, Machine learning, Image processing, Cellular imaging

## Abstract

Machine learning-based systems have become instrumental in augmenting global efforts to combat cervical cancer. A burgeoning area of research focuses on leveraging artificial intelligence to enhance the cervical screening process, primarily through the exhaustive examination of Pap smears, traditionally reliant on the meticulous and labor-intensive analysis conducted by specialized experts. Despite the existence of some comprehensive and readily accessible datasets, the field is presently constrained by the limited volume of publicly available images and smears. As a remedy, our work unveils APACC (**A**nnotated **PA**p cell images and smear slices for **C**ell **C**lassification), a comprehensive dataset designed to bridge this gap. The APACC dataset features a remarkable array of images crucial for advancing research in this field. It comprises 103,675 annotated cell images, carefully extracted from 107 whole smears, which are further divided into 21,371 sub-regions for a more refined analysis. This dataset includes a vast number of cell images from conventional Pap smears and their specific locations on each smear, offering a valuable resource for in-depth investigation and study.

## Background & Summary

Cervical cancer remains the most common type of cancer among women to date. According to Cohen *et al*.^1^, half a million women are diagnosed with cervical cancer in every year, and more than 300,000 cases are fatal. Although the use of an increasingly advanced version of Pap smear screening can reduce severe outcomes in many cases^2^, the difficulty of accessing and performing the diagnostic test, especially in less economically developed countries, makes the problem even more significant^3^. The main problem resides in the expensive nature of the conventional procedure, combined with it being highly labor-intensive, and requiring multiple expert specialists to conduct efficiently while also assuring the highest standards of quality. To decrease the costs of the examination and quality assurance, and ultimately reduce fatal outcomes, multiple different methods have been developed, where AI-based solutions have been introduced in addition to traditional methods^4^.

Although public datasets are becoming more and more accessible, there are cases, where machine learning-based systems are trained using a private dataset^5–7^. This is perhaps because producing an annotated dataset for training a reliable system is an extremely time-consuming and expensive process, as it requires the collaboration of many highly skilled experts, often over up to several years. In cases where it is not possible to produce a private dataset, researchers are forced to rely on publicly available ones. In various instances, this can cause difficulties, as the currently available datasets are highly limited, especially in terms of the number of annotated cell images extracted from smear slices. In the related literature, we could locate three different public datasets, which are the Herlev^8^, SIPaKMeD^9^, and CRIC Cervix^10^.

The Herlev dataset is the oldest of the three, released in 2005 to aid the development of further classification systems. Unfortunately, the dataset is very small in number, with a total of 917 cell images. The cells were classified into seven classes: Squamous cell carcinoma *in situ* intermediate (SCCIS), Severe squamous non-keratinizing dysplasia (SSNKD), Moderate squamous non-keratinizing dysplasia (MSNKD), Mild squamous non-keratinizing dysplasia (MiSNKD), Columnar epithelial (CE), Intermediate squamous epithelial (ISE), and Superficial squamous epithelial (SQE). The images were annotated by two cyto-technicians, as well as a cytopathologist in difficult cases. When there was no consensus among the experts, the sample was discarded. There is no information on how many different smears the cell images were obtained from in total. One positive aspect of the dataset is that it contains more abnormal, rarely occurring cells than normal ones. The paper also describes the performance of basic classification systems, which are now significantly outperformed by more modern systems, but these usually require more data to learn.

The newer SIPaKMeD dataset published in 2018 is significantly larger than Herlev. It contains a total of 4,049 cell images extracted from 966 smear slices. The exact number of whole smears from which the data was extracted is not provided. The cell images were classified by expert cytopathologists into the following five classes: Parabasal (PARA), Superficial-intermediate (SI), Dyskeratotic (DYSK), Koilocytotic (KOIL), and Metaplastic (META). The dataset has the advantage that the sample distribution among the classes is almost balanced, so it can be easily trained by machine learning-based models.

The CRIC Cervix dataset is the latest and most advanced one currently available. It contains a total of 11,534 different cell images, which have been classified into six classes according to the Bethesda System nomenclature^11^: negative for intraepithelial lesion or malignancy (NILM), atypical squamous cells of undetermined significance that are possibly non-neoplastic (ASC-US), low-grade squamous cell carcinoma (SCC) intraepithelial lesion (LSIL), atypical squamous cells that cannot exclude a high-grade lesion (ASC-H), high-grade squamous intraepithelial lesion (HSIL), and squamous cell carcinoma (SCC). The annotation was performed by three cytopathologists. The dataset has the advantage of being interactively accessible via a web application and can be used to retrieve individual cell images, smear slices, and cell locations. The authors point out that in several cases the images in the previously created public datasets are almost too “clean”, which can indicate that a rigorous pre-filtering of the images was probably performed before publication. This can be a disadvantage when developing an automated system if the same type and level of pre-filtering is not feasible.

In this paper, we present a new public dataset, which was gathered in the framework of a research and development project in collaboration among experts from the Department of Pathology of the Clinical Center, and the Faculty of Informatics of the University of Debrecen. The APACC dataset^12^ contains a total of 103,675 cell images extracted from 107 whole smears (from the same number of patients), that were divided into 21,371 smaller (2,000 × 2,000) smear slices. During the extraction of the cell images, there was no pre-filtering involved. The cell images were segmented automatically using a deep learning-based system; for more details see the Methods section. The extracted cells were divided equally among three cytopathologists on a smear basis, followed by a random shuffling. There was no overlap regarding the annotation of the cell images between the medical experts. The cytopathologists annotated each cell individually, however, the more difficult cases, where the single expert could not indubitably identify the appropriate class, were separated for a second assessment. These cases were then discussed during a counsel among the three experts, where they reached a consensus about the final annotation of these images.

A numerical comparison between the currently available public datasets and our dataset is presented in Table 1. The APACC dataset^12^ is the largest one, with 103,675 cell images in total. It was composed simultaneously with the development of an automatic screening system, so the main goal was to create the most suitable dataset for the development of such machine learning-based solutions. The most noteworthy advantage of APACC over the currently available datasets is the large amount of images. Furthermore, the dataset is split into train and test subsets, which enables a uniform evaluation for different approaches. It is also worth mentioning that the images in the dataset were not manually selected based on their ease of processing. What the system automatically extracted was annotated in the same form as it originally appeared in the smear. In our opinion, the APACC dataset^12^ could serve as a basis for many new research projects and could also help in the evaluation of existing systems, possibly as a new benchmark dataset that could be widely applied.

**Table 1:** Comparison of the publicly available datasets with the proposed APACC one.

Attribute	Herlev	SIPaKMeD	CRIC Cervix	APACC
Number of smears used	—	—	—	107
Number of smear slices	—	966	400	21,371
Number of cell images	917	4,049	11,534	103,675
Number of classes	7	5	6	4
Annotated by	2 cyto-technicians (+1 doctor)	expert cytopathologists	3 cytopathologists	3 cytopathologists

## Methods

The research conducted at the University of Debrecen, Debrecen, Hungary, received approval from the Scientific and Research Ethics Committee of the Health Sciences Council of Hungary, referred to later as IRB, under protocol number OGYÉI/65989/2020. The data collected from anonymous samples preclude the identification of patients. Consequently, participants were not obligated to provide consent for data sharing. The IRB has waived and delegated the authority to publish and approve this work to the project leaders, namely Prof. Dr. Andras Hajdu and Dr. Ilona Kovacs. In accordance with the agreements outlined above, publication approval necessitates the endorsement of at least one of these project leaders, adhering to the ethical standards set forth in the World Medical Association’s Helsinki Declaration and the University of Debrecen’s scientific application regulations for ethical requirements in scientific publications.

Traditional Pap smear procedures were executed by extracting cells from the cervix’s squamocolumnar junction, employing specialized apparatuses such as the Cervex-Brush or Cyto-Brush. Following the extraction, these cellular specimens were methodically allocated onto microscopic slides and instantaneously preserved using a 95% ethyl alcohol medium or an alternative spray fixative. To enable distinctive cytological scrutiny, each specimen was subjected to a staining regimen utilizing the Papanicolaou stain, succeeded by an exhaustive assessment by expert cytologists, achieving an annotation of the cell images performed individually without overlaps between three cytopathologists, employing the fine classifications articulated in the Bethesda 2014 framework.

Negative as well as abnormal smears were used in compiling the dataset. In the case of negative samples, only interpretability was taken into account during the selection, meaning that the technically unsuitable ones were not utilized. Following the analysis of abnormal smears, a positive histological examination was a prerequisite for selection: CIN2 or more severe cervical intraepithelial neoplasia.

**Fig. 1:**
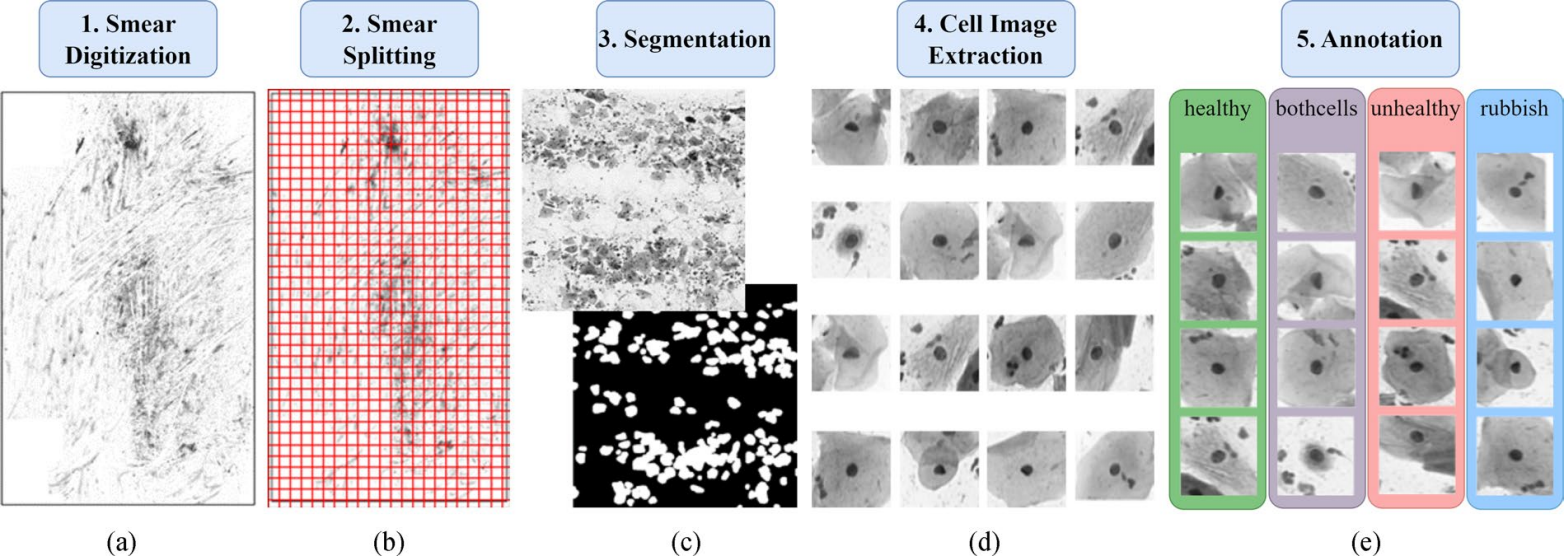
The whole data extraction process, where (**a**) is the smear to be digitized, (**b**) represents the splitting of the whole smear into smaller sections, (**c**) shows a single split and the resulting binary mask, (**d**) depicts example individual cell images extracted using the segmentation mask, and (**e**) illustrates the annotation process done by medical experts.

Following the clinical extraction process and the selection of smears, the dataset is built by following a 5-step process consisting of the digitization of the smears, the slicing of them, segmentation of cell groups, detailed cell image extraction, and finally the manual annotation of the cells. This process is also illustrated in Fig. 1.

### Smear Digitization

The smears are scanned using a 3DHistech Pannoramic 1000 scanner with an Adimec Q-12A-180Fc brightfield camera. The scanner creates images with three different focus setups (using 3 micrometers step between them), then selects the sharpest layer from each focus level for each image field and combines them into a single layer^13^. A 20x microscope objective is used for scanning, resulting in a digitized image with about 100,000 × 220,000 pixels as shown in Fig. 1(a). These images are compressed and saved in a special MRXS format including multiple resolution levels. To find specific regions of interest, an area to locate cells is identified at the lowest resolution level using intensity values. This setup allows a detailed examination of cells, including their nuclear chromatin distribution. It ensures that different patterns are visible in the digital images, and cell groupings can be easily identified.

### Smear Splitting

After the digitization of the smear, it is necessary to split the digitized smears to achieve efficient data processing. Since the overall size of a smear is exceptionally large, it is divided into smaller slices of 2,000 × 2,000 pixels as seen in Fig. 1(b). The automatic splitting of the smears is done by first extracting the FOV from the lowest resolution level of the image using the intensity values, followed by the decomposition process to achieve non-overlapping slices. These procedures, and the total magnification level of 200x (combination of the 20x magnification lens and the 10x eyepiece) allow the examination of individual cells as well as cell groups with appropriate detail. The resulting smear slices are sufficiently small to be processed with machine learning algorithms and comfortably fit into GPU memory.

### Segmentation

The extracted smear slices are processed using a neural network-based algorithm, which can segment all cell regions in the slices. Our proposed method uses the fully convolutional network (FCN) ensemble presented in an earlier paper^14^ to perform the segmentation. The essence of the method is to combine the results computed by different FCNs together with the original input images, which results in better performance compared to other state-of-the-art solutions. The goal with the application of this system is to create a binary mask (see Fig. 1(c)) for each 2,000 × 2,000 smear slice, where white pixels represent cell regions and black pixels represent the background ones.

### Cell Image Extraction

**Fig. 2:**
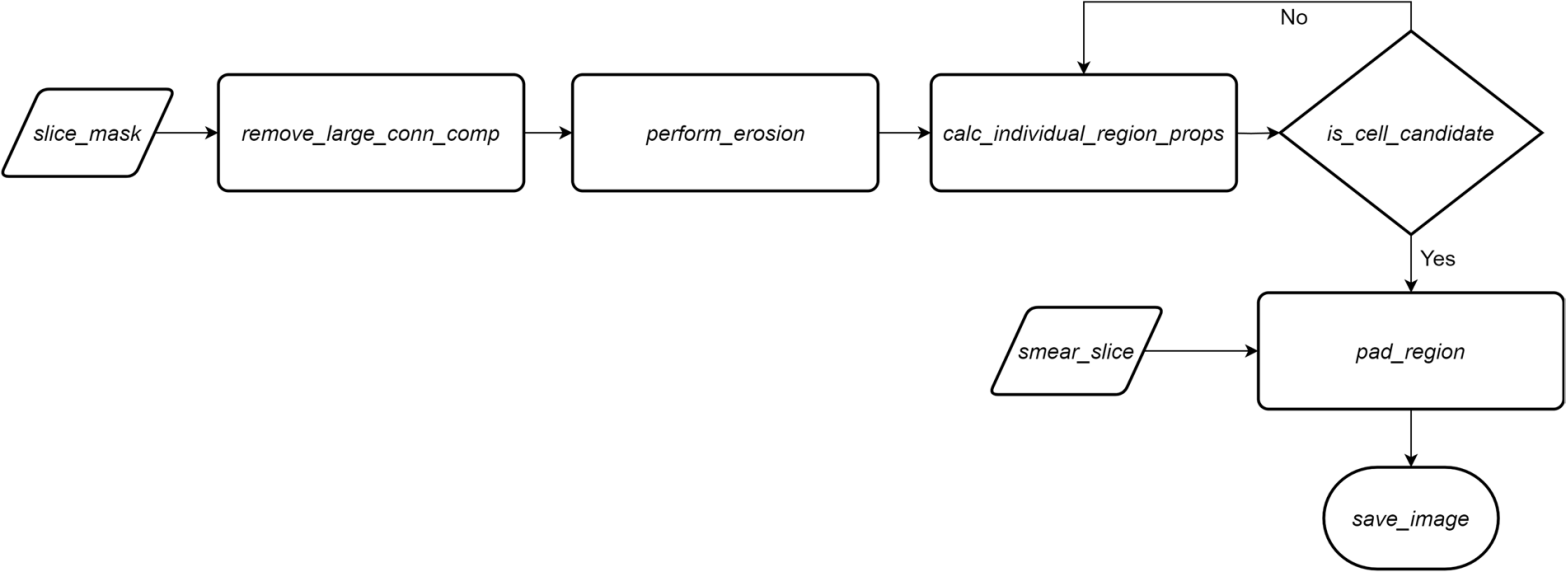
The steps of the cell extraction procedure.

Using a combination of the computed binary masks and original input images, an algorithm capable of extracting individual cells is developed; for some outputs see Fig. 1(d). The cell extraction starts by reading the respective binary mask (*slice_mask*) and removing every large connected component from the image based on a previously defined threshold (*remove_large_conn_comp*). Next, multiple erosions are performed on the remaining regions with the goal to maximise the number of individual cell counts on the images (*perform_erosion*). For all individual components the appropriate region properties are calculated (*calc_individual_region_props*). Based on the area and the roundness of the region, cell candidates are selected (*is_cell_candidate*). In case of a candidate, the region is located on the original smear slice (*smear_slice*), then padded in each direction (*pad_region*), and finally exported as a cell image (*save_image*). This process is illustrated in Fig. 2. Using this method, smaller images containing potential cell images are extracted efficiently with the exact size depending on the particular cell.

### Annotation

The last step was the annotation, where the cytopathologists marked the cells/cell groups extracted from the provided digitized sample. A simple IT solution was implemented that is capable of loading the image files containing the cells to be annotated directly from a network drive, enabling the examination process to be done efficiently. The result images can contain either individual cells or cell conglomerates, especially in cases where there is an overlap between the cells. The annotation process involves carefully inspecting the automatically loaded images to determine if the cells and their surroundings are in a healthy state, exhibiting signs of abnormalities, or if the image does not contain interpretable cells. In some cases, the image can show a mix of both healthy and unhealthy cells. By following this procedure, all extracted images are classified into four distinct categories, as illustrated in Fig. 1(e).

**Fig. 3:**
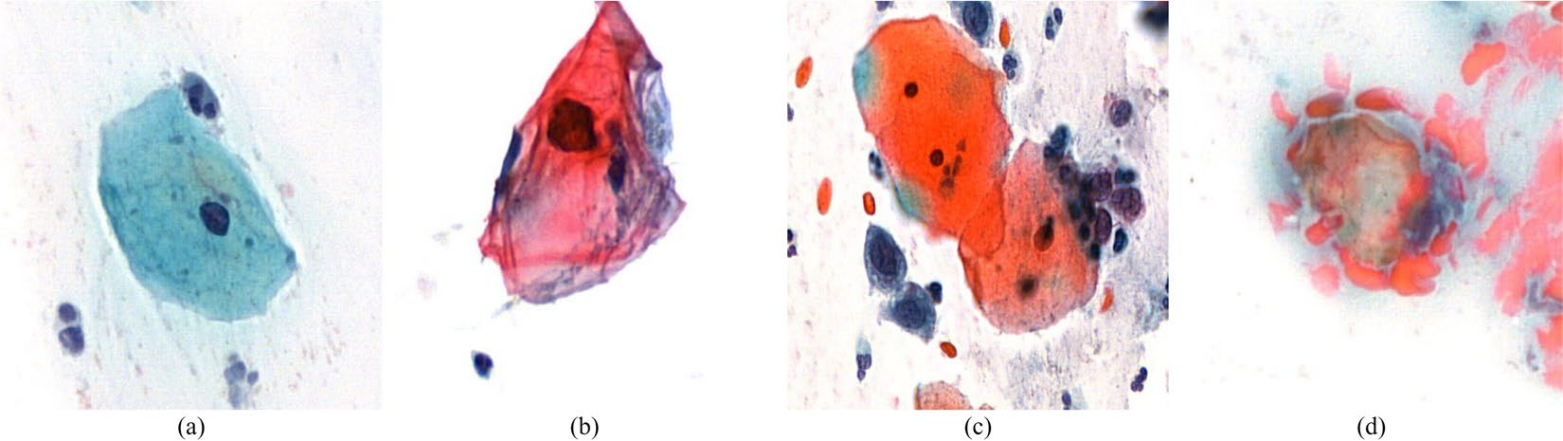
Example cell images, where the image is labeled as (**a**) healthy, (**b**) unhealthy, (**c**) bothcells, and (**d**) rubbish.

Once the annotation is complete, the result is also saved to the network drive, from where it can be used to build the training database. Using this process, 103,675 images have been annotated by cytopathologists for image recognition algorithms. Thus, the experts performed the annotation of the extracted cells/cell groups, classifying them as healthy (normal), unhealthy (abnormal), rubbish (not valid), and bothcells (both healthy and unhealthy cells are present). To give an impression of these classes and the categories they represent in the Bethesda system, we list them as follows. The healthy class represents cells from the Negative for intraepithelial lesion or malignancy (NILM) category. The unhealthy class contains cells from the Epithelial cell abnormality Bethesda category, where there was no additional sub-division into the ASC, LSIL, and HSIL categories, the class containing cells from each of these. The rubbish class represents the Unsatisfactory for evaluation Bethesda category. The bothcells class also represents the Epithelial cell abnormality Bethesda category, since it contains malignant cells, however, these images also contain more healthy cells. Examples of images from the four classes are depicted in Fig. 3.

During the manual annotation process, a randomly selected subset of an equal number of samples was annotated by each cytopathologist. In case of uncertainty, they consulted with each other, and the consensus opinion was the result of the evaluation.

## Data Records

This section gives a detailed description of the APACC dataset^12^ that has been deposited to the Open Science Framework (OSF) platform. The sizes of its cell images vary, depending on the original sizes of the cells. However, the sizes of the cell images are relatively small including only the related cell/cell groups. Particular care has been taken to ensure that cell surroundings remain visible in the images, as this may affect the classification of a cell into a certain class. The images contain all information of their origin in their names, thus a precise localization is available. The naming follows the format *{smear id}_{smear slice location}_{cell number}_{cell location}_{date}.png*. For example, the cell image named as *669-15_36000-74000_10_850-1300_2021-07-07.png* is extracted from the smear having the id *669-15*, the location of the smear slice is *36000-74000* representing a coordinate pair pointing to the left-top corner of the extracted area, the number of the cell is *10*, the location of the cell on the particular smear slice is *850-1300*, representing a coordinate pair pointing to the center of the cell image, and the date of extraction is *2021-07-07* (in a *%Y-%m-%d* format).

**Fig. 4:**
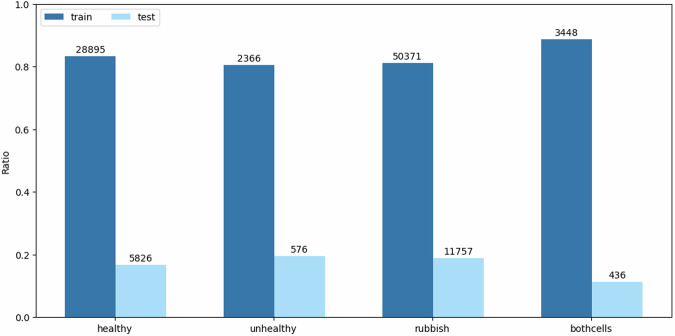
Class distributions regarding the training and test subsets.

The total number of cell images is 103,675, which were extracted from 21,371 smear slices of 107 whole smears belonging to individual patients. In terms of classes, this means 34,721 healthy, 2,942 unhealthy, 62,074 rubbish, and 3,884 bothcells images. The distribution of the classes among the smear slices is key information we use when considering splitting the dataset^12^ into training and testing subsets. 20 smears are selected for the test set such that the distribution of the classes for the two subsets coincides with the original as much as possible. The class distributions are also illustrated in Fig. 4. Details about the quantities of images used from each class in the respective subsets are presented in Table 2, where the number of smears, smear slices, and cell images can be viewed, following the train and test splits.

During the clinical evaluation of cells, the cellular environment is important. The extracted images include this environment based on the rules defined during annotation, but there is also the possibility for the precise localization of the cells. As such, we include further information about the original location of each cell on its corresponding 2,000 × 2,000 smear slice. For each smear slice, a text document is also provided, where one record represents a cell located on that particular smear slice. In each record, the class of the cell is mentioned followed by the horizontal and vertical coordinates of the center, the width, and the height of the cell, normalized to fall between 0 and 1. Using these data, one can decide to explore the localization of the different cell images in the smears. In addition to this, we select 34 whole slide images where the number of annotated cell regions are the highest, and we include these into the dataset as well.

The APACC dataset^12^ was made publicly available under the Creative Commons Attribution 4.0 International Public License on the Open Science Framework (OSF) platform. We provide an additional GitHub repository for ease of use. Please find additional details in the Usage Notes and Code Availability sections.

**Table 2:** Overview of the APACC dataset.

Dataset	Smear	Smear slice	healthy cells	unhealthy cells	rubbish cells	bothcells
Train	87	17,387	28,895	2,366	50,371	3,448
Test	20	3,984	5,826	576	11,757	436
Total	107	21,371	34,721	2,942	62,074	3,884

## Technical Validation

In this section, we show an example of a real-world problem that can be solved using deep learning models that are currently relatively easy to obtain and apply in combination with the images in the APACC dataset^12^. In essence, we provide a direction for further research and a baseline sample performance value.

The dataset can be mainly used to solve the problem of cell classification in conventional Pap-smear images. There are many clinical challenges in the literature where the investigated problem can be solved by an automated system capable of classification^15^. However, in such a case, the basis of generally well-performing systems is usually a neural network^16^. We demonstrate the usefulness of the dataset in such cases by training several neural networks with different architectures in the same way and comparing their performance. This gives us an idea of which architecture might be the right choice for the problem in question, as well as a reasonably detailed overview of what performance we can expect in general when using such a system.

The classes presented previously are defined as the labels to be predicted by the models. That is, in the deep learning process, the ability to classify images of cells into healthy, unhealthy, rubbish, and bothcells is trained. First, 15% of the images in the training set are separated as a validation set, keeping the original distributions among the classes.

Simple applicability is a primary consideration in the choice of the architectures, as we aim to give a standard that can be a starting point for future research. Accordingly, the following architectures have been selected: DenseNet-121^17^, DenseNet-201^17^, EffNet-B3^18^, EffNet-B5^18^, EffNet-B6^18^, NasNet-Large^19^, ResNet-50^20^, Inception-V3^21^, and InceptionResNet-V2^22^. In all cases, the input layer is set to be able to process images of size 224 × 224 × 3, and the cell images were symmetrically padded with white pixels to achieve this dimension. This is an important step, since using this method, we do not lose the original proportions of the cells, and avoid inserting extra dissimilarities between the cell images, since the original smear background is also white. Furthermore, at the end of the neural architectures, we add a GlobalAveragePooling2D layer, a fully connected layer of 512 neurons (with ReLU activation function), a Dropout layer with probability value 0.5, and an output layer of 4 neurons (with Softmax activation function). Training is performed over 30 epochs using ADAM optimizer starting with a learning rate of 1e-3 and decreasing during training using the ReduceLROnPlateau tool based on the loss value obtained on the validation set with a patience parameter of 3 and a factor of 0.05. In addition, we also use an EarlyStopping callback function, which can stop learning earlier based on the validation set if the loss value does not decrease significantly for at least 6 epochs. The batch size values are set to the largest possible one considering the used hardware, ranging from 8 to 128 in the different models. To avoid overfitting, several augmentation techniques, including flipping, zooming, and rotation, are applied. These methods also adhere to the important requirement of maintaining the ratio between the nucleus and cytoplasm.

The individual models are evaluated on the test dataset. To measure the performance comprehensively, F1-Score weighted by the number of samples from different classes, Accuracy, and ROC/AUC metrics are calculated. The precise results for each model are presented in Table 3. It can be observed that the variation in the performance among the individual models is not significant. Furthermore, it is noticeable that a high number of parameters does not significantly enhance the performance. The model achieving the best performance is EfficientNet-B3, which is relatively small based on the number of parameters, making it a promising base architecture.

**Table 3:** Classification results for various models and metrics on the test dataset.

Model name	Accuracy	F1-Score	ROC AUC	Parameters
DenseNet-121	0.8144	0.8100	0.8865	7.6M
DenseNet-201	0.8158	0.8113	0.8835	20.2M
**EfficientNet-B3**	**0.8228**	**0.8204**	**0.9009**	**12.0M**
EfficientNet-B5	0.8190	0.8087	0.8925	30.0M
EfficientNet-B6	0.8215	0.8167	0.8884	43.0M
NasNet-Large	0.8087	0.7886	0.8404	89.0M
ResNet-50	0.8107	0.7994	0.8795	26.0M
Inception-V3	0.8155	0.8052	0.8816	24.0M
InceptionResNet-V2	0.8177	0.8093	0.8959	56.0M

It is important to point out that during the testing process, we observed that the network performance on the class with the lowest number of images is low compared to the overall performance. This might indicate that the main difficulty is the imbalanced nature of the dataset. Within the scope of this paper, we do not aim to solve this problem, but we suggest how to make the dataset more balanced. One example is the use of images of the class bothcells as unhealthy ones. Since these images include examples from both healthy and unhealthy classes, we can assume that the learning process will not be driven in the wrong direction, and in return, we eliminate the class with the smallest number of cells. Overall, we can claim that one of the main challenges in developing an automatic screening system capable of classifying given cell images into different classes is the imbalance in the dataset.

## Usage Notes

The APACC dataset^12^ is made public under the Creative Commons Attribution 4.0 International Public License using the Open Science Framework (OSF) platform and is available at https://osf.io/fp2xe. A sample is available containing the first 100 images from every dataset and category. The full dataset is also available uploaded into three folders. One of them contains the cell images, split into training and test sets, and grouped by their classes. The second folder contains the smear slices, following the same training-test split, and containing also a text document for each smear slice, where one can find information about each annotated cell location. In addition, in the third folder, those 34 whole slide images are provided, that contain the most amount of annotated cells.

**Fig. 5:**
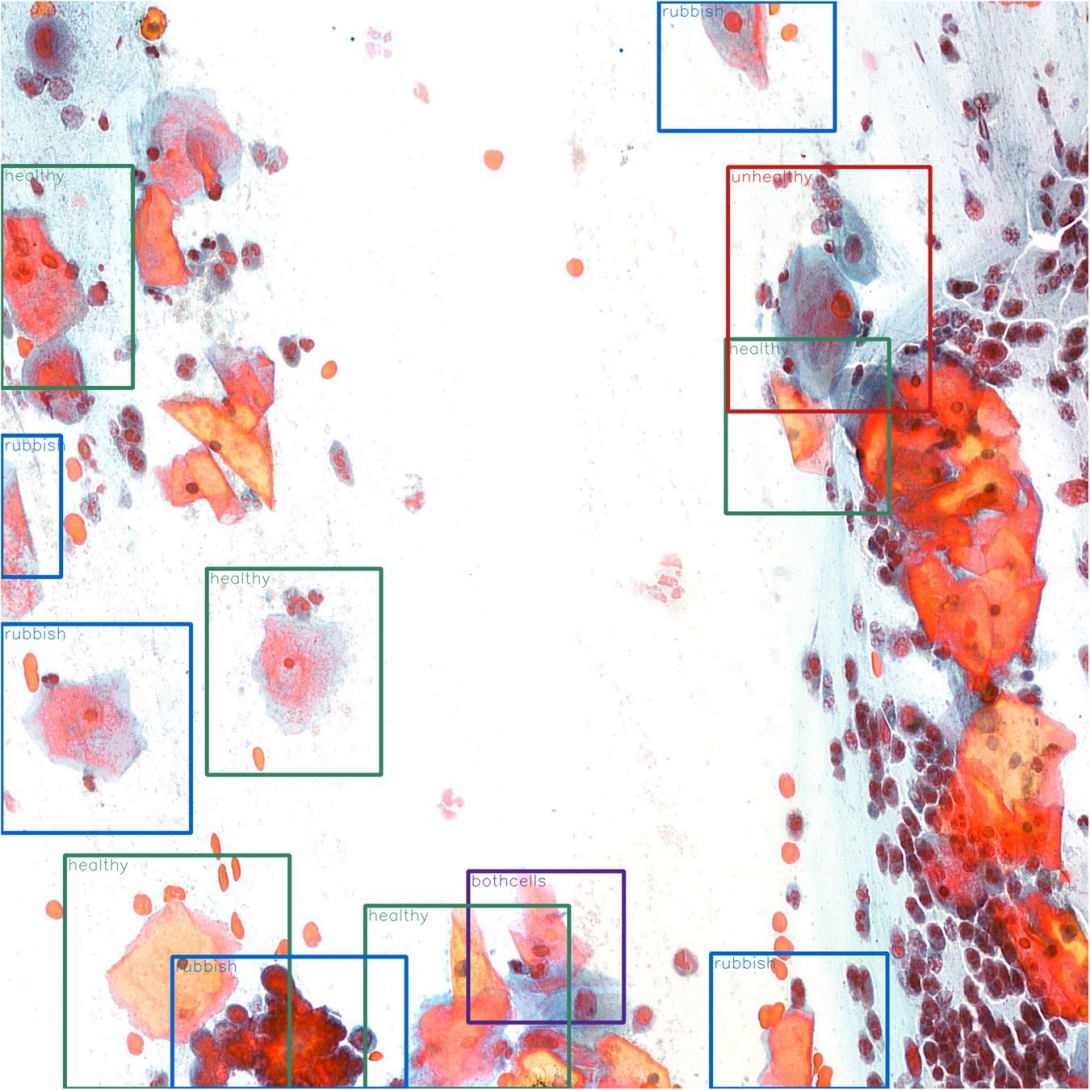
An example for an annotated smear slice.

Along with the main dataset, we provide a collection of assisting source codes. Primarily, the code made available can help in reconstructing the cell locations on their original smear slice, using the provided text documents. By using this software, annotated smear slices can be constructed, where each labeled cell on the slice is indicated by colored borders. The true class of each cell is indicated by color (healthy - green, rubbish - blue, unhealthy - red, and bothcells - purple) to make them easy to identify. In addition, the name of the class is also shown for each labeled area; an example image can be observed in Fig. 5.

## Data Availability

The source code is available at https://github.com/david-kupas/apacc-smear-cell-db and can be publicly accessed under the GNU General Public License v3.0. The exact details of the usage can also be accessed through the link provided, accompanied by example codes. The code was written in Python language using the NumPy, OpenCV, Pillow, Matplotlib, and Scikit-Image packages.
